# Supplementary material for: Accurate genome-wide genotyping from archival tissue to explore the contribution of common genetic variants to pre-cancer outcomes
Source: J Transl Med. 2022 Dec 27;20:623. doi: 10.1186/s12967-022-03810-z (PMC9793518; doi:10.1186/s12967-022-03810-z)
Supplement: Supplementary file 1 — Additional file 1: Figure S1. Effect of patient ancestry on lc-WGS imputation concordance between blood and tissue. Comparison of concordance between blood and tissue-based on ancestry background of the patient, with White ancestry in light green and Black or Asian ancestry in dark green. Pearson correlation squared (r2) is for all aggregated SNPs within a MAF bin. When available, 95% confidence intervals are shaded around the line based on 1000 bootstrap iterations. Figure S2. Effect of coverage-related features on lc-WGS imputation concordance between blood and tissue. (a, b) Comparison of concordance as measured by squared Pearson correlation (y-axis) between blood and tissue as a function of MAF (x-axis) based on mean sequencing genome coverage depth of blood (a) or tissue (b). Figure S3. Effect of coverage-related features on the error in non-cancer PRS calculation. (a, b) PRS error (y-axis), as measured by the absolute difference between blood and tissue PRS across all non-cancer PRS, as a function of (a) fraction of genome in a copy number loss or (b) mean tissue/tumor genome coverage (x-axis). The 95% confidence intervals are shaded around the line based on 1000 bootstrap iterations. Spearman correlation coefficient, r, and corresponding p-value are indicated as text in the upper right. Figure S4. Cox proportional hazard models measuring BCSE outcome in DCIS patients for 6 breast cancer PRS. Forest plot representation of hazard ratios (square) and 95% confidence intervals (error-bars), for each normalized breast cancer PRS and covariates for DCIS BCSE risk including DCIS nuclear grade (Grade), age of the patient at diagnosis (Age), the size of the DCIS lesion (Size), and whether the ancestry of the individual was European (EUR). The dotted line represents a hazard ratio of 1, indicating no effect on BCSE risk, > 1 indicating increased, and < 1 indicating decreased risk. Figure S5. Assessment of 2 field HLA-typing accuracy from lc-WGS. Number of concordant HL [file 12967_2022_3810_MOESM1_ESM.docx]

## Additional file 1

### Figure S1. Effect of patient ancestry on lc-WGS imputation concordance between blood and tissue. Comparison of concordance between blood and tissue-based on ancestry background of the patient, with White ancestry in light green and Black or Asian ancestry in dark green. Pearson correlation squared (r^2^) is for all aggregated SNPs within a MAF bin. When available, 95% confidence intervals are shaded around the line based on 1000 bootstrap iterations.
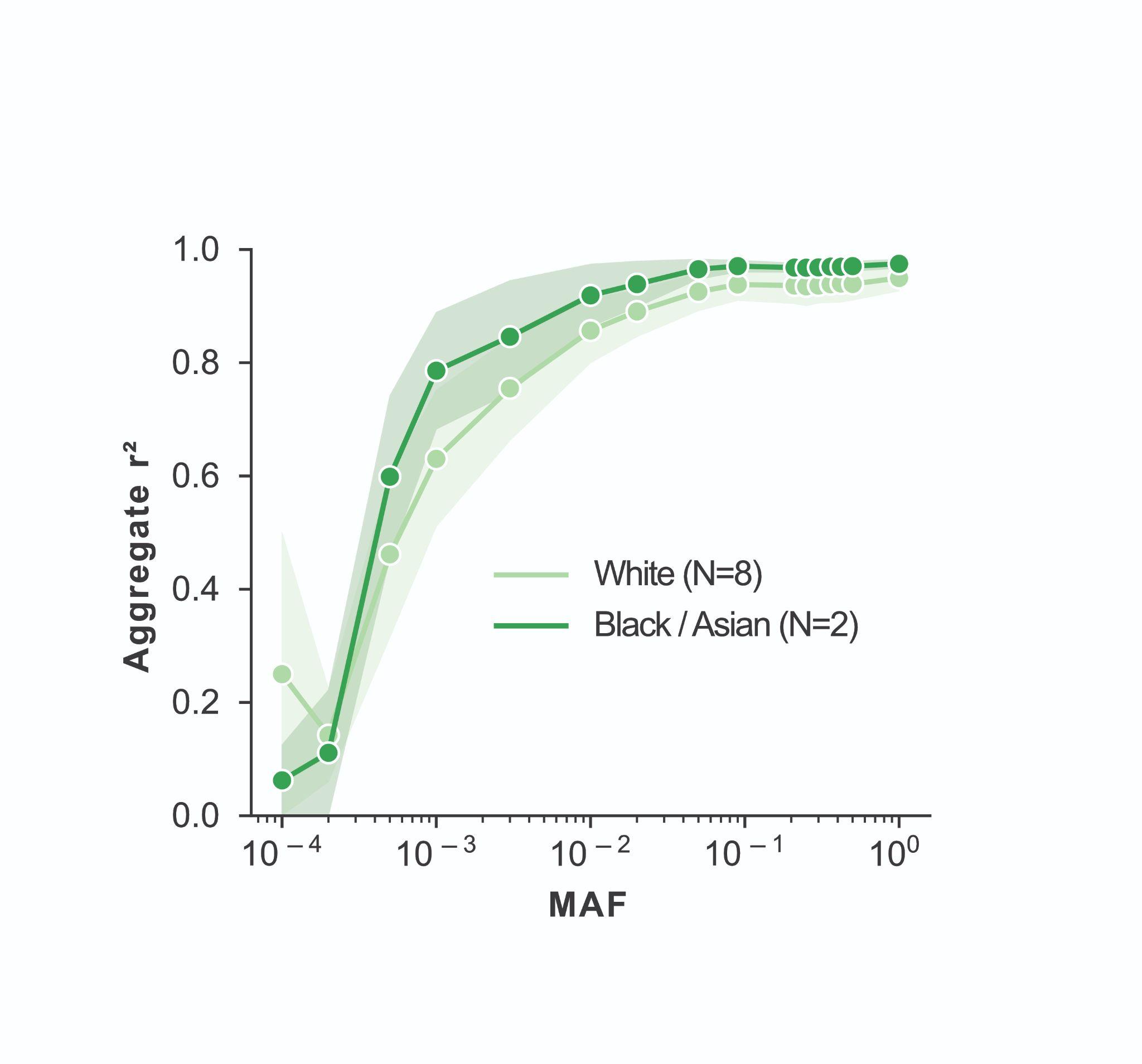


###

###

###

###

###

###

### Figure S2. Effect of coverage-related features on lc-WGS imputation concordance between blood and tissue. (a,b) Comparison of concordance as measured by squared Pearson correlation (y-axis) between blood and tissue as a function of MAF (x-axis) based on mean sequencing genome coverage depth of blood (a) or tissue (b).
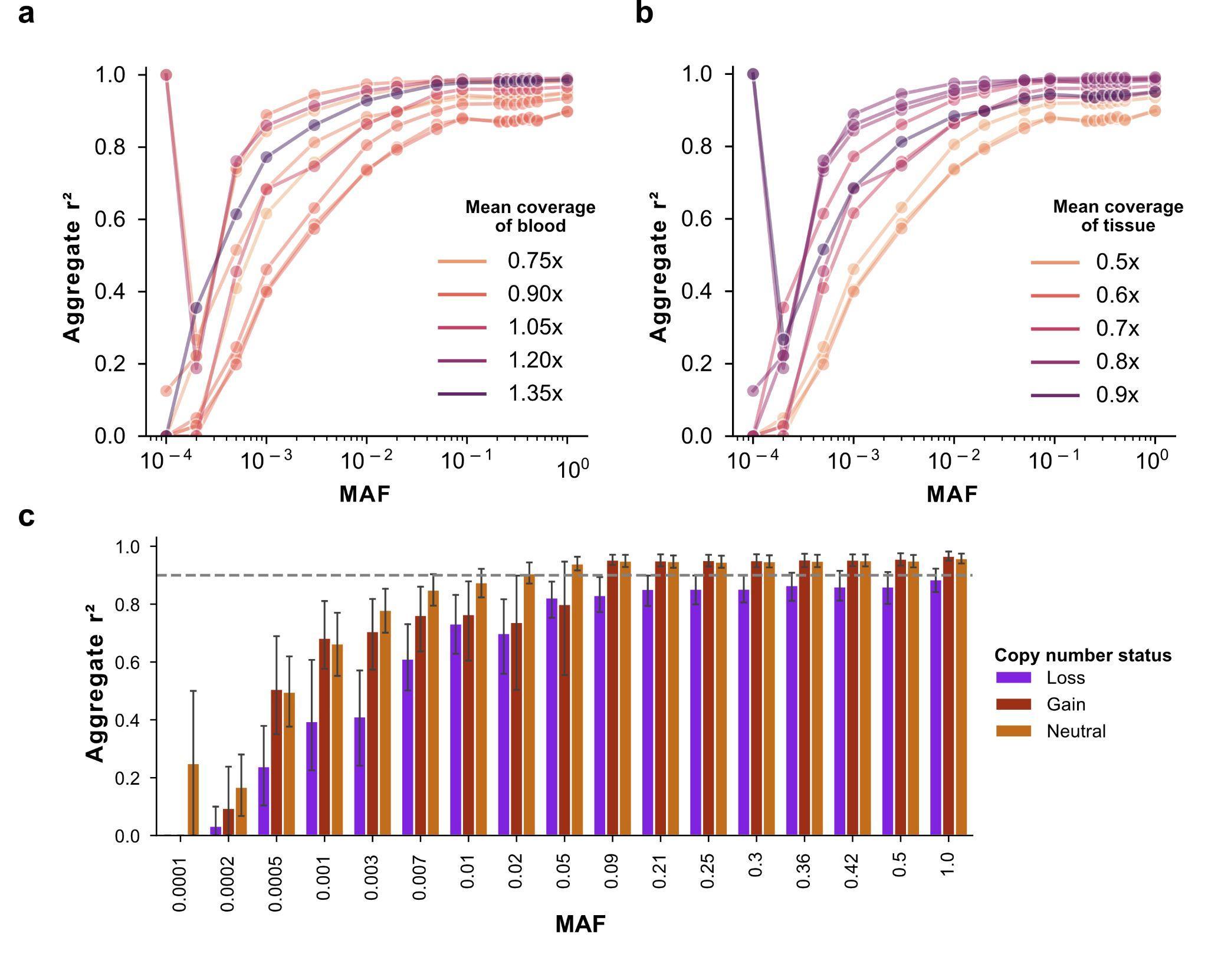


###
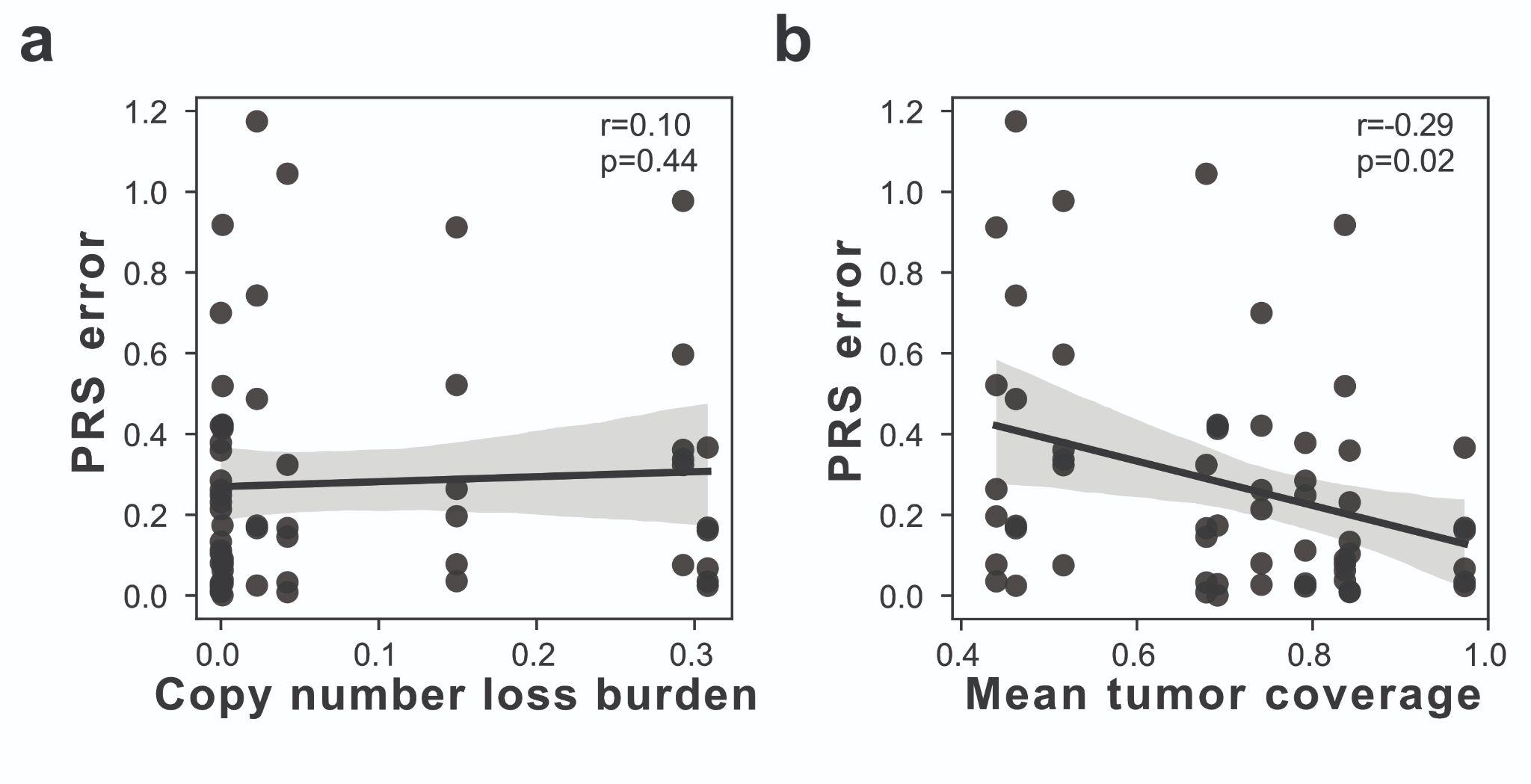
Figure S3. Effect of coverage-related features on the error in non-cancer PRS calculation. (a-b) PRS error (y-axis), as measured by the absolute difference between blood and tissue PRS across all non-cancer PRS, as a function of (a) fraction of genome in a copy number loss or (b) mean tissue/tumor genome coverage (x-axis). The 95% confidence intervals are shaded around the line based on 1000 bootstrap iterations. Spearman correlation coefficient, r, and corresponding p-value are indicated as text in the upper right.

###

###

###

###

###

###

###

###

###
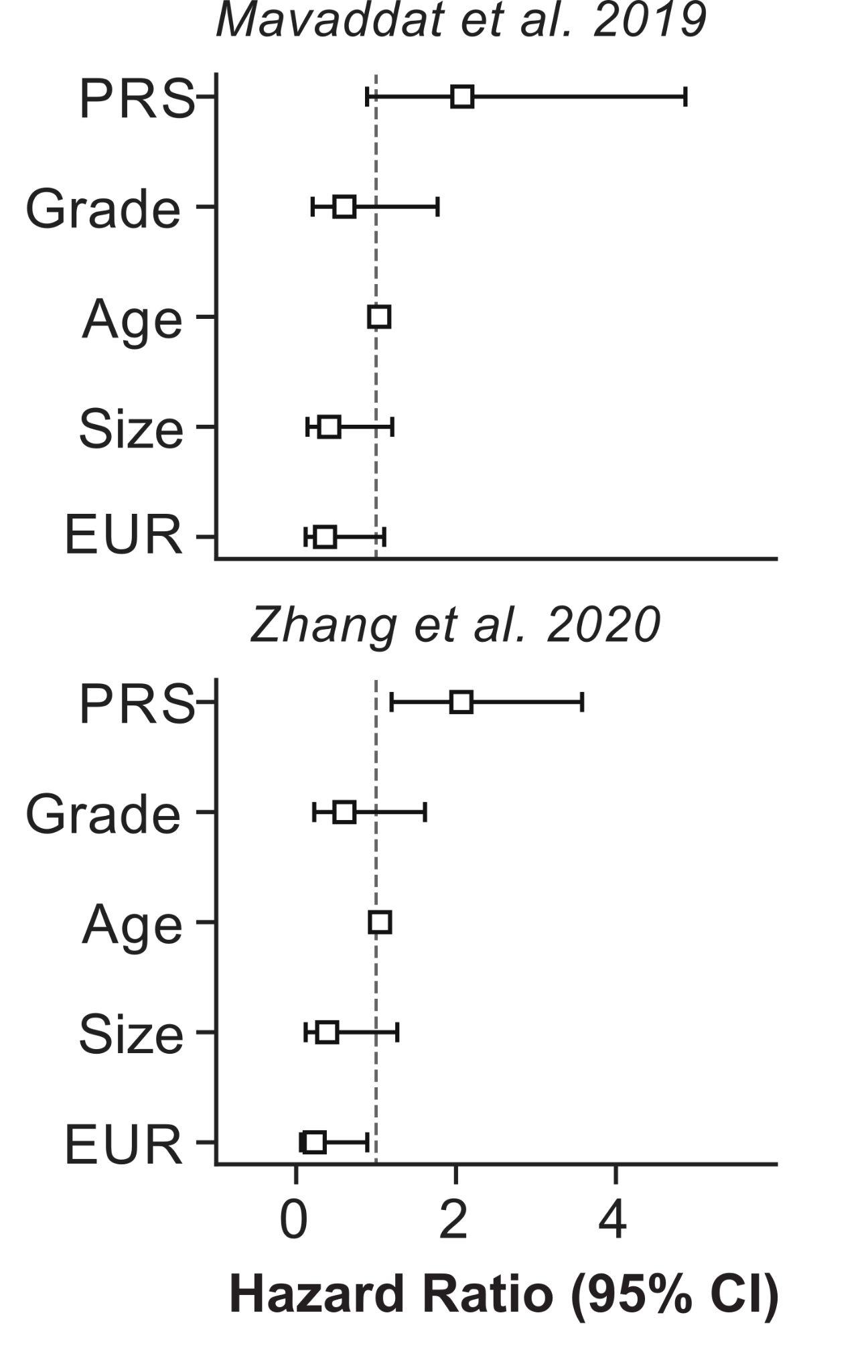


### Figure S4. Cox proportional hazard models measuring BCSE outcome in DCIS patients for 6 breast cancer PRS. Forest plot representation of hazard ratios (square) and 95% confidence intervals (error-bars), for each normalized breast cancer PRS and covariates for DCIS BCSE risk including DCIS nuclear grade (Grade), age of the patient at diagnosis (Age), the size of the DCIS lesion (Size), and whether the ancestry of the individual was European (EUR). The dotted line represents a hazard ratio of 1, indicating no effect on BCSE risk, >1 indicating increased, and <1 indicating decreased risk.

###

###
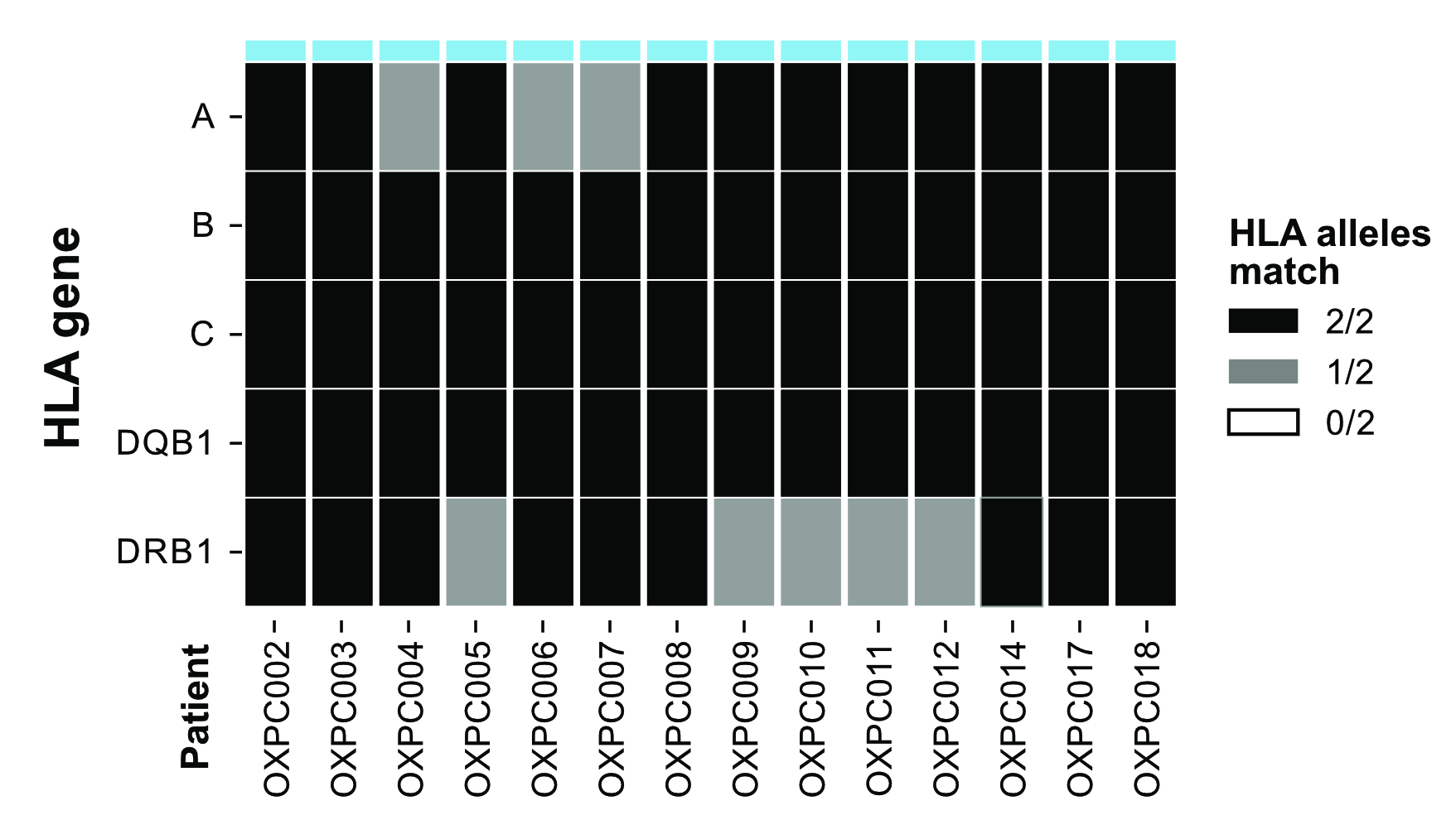
Figure S5. Assessment of 2 field HLA-typing accuracy from lc-WGS. Number of concordant HLA alleles (0: white, 1: grey:, 2: black) between haplotypes from the clinical gold standard and those imputed using QUILT-HLA for class I (A, B, C) and class II (DQB1 and DRB1) HLA genes (rows) using blood DNA of 14 patients.

###

###

### Table S1. Description of the studied samples.

| **Patient** | **DNA source** | **Ancestry** | **Input DNA (ng)** | **Coverage** | **CNA burden** | **Age of block (yr)^1^** | **Analysis^2^** |
| --- | --- | --- | --- | --- | --- | --- | --- |
| OXPC002 | Blood | White | 68.4 | 1.11 | 0.00 | - | H |
| OXPC003 | Blood | White | 31.8 | 1.41 | 0.00 | - | GPH |
| OXPC003 | Tissue | White | 28.4 | 0.69 | 0.00 | 7 | GPH |
| OXPC004 | Blood | White | 32.1 | 0.84 | 0.00 | - | GPH |
| OXPC004 | Tissue | White | 5.5 | 0.44 | 0.15 | 7 | GPH |
| OXPC005 | Blood | White | 53.1 | 0.79 | 0.00 | 5 | GPH |
| OXPC005 | Tissue | White | 21.8 | 0.97 | 0.48 | - | GPH |
| OXPC006 | Blood | White | 48.9 | 0.95 | 0.00 | - | H |
| OXPC007 | Blood | White | 54.6 | 0.87 | 0.00 | - | GPH |
| OXPC007 | Tissue | White | 35.1 | 0.46 | 0.08 | 6 | GPH |
| OXPC008 | Blood | White | 45.3 | 0.89 | 0.00 | - | H |
| OXPC009 | Blood | White | 12.2 | 1.10 | 0.00 | - | GPH |
| OXPC009 | Tissue | White | 11.0 | 0.84 | 0.01 | 5 | GPH |
| OXPC010 | Blood | Black or African American | 16.9 | 0.85 | 0.00 | - | GPH |
| OXPC010 | Tissue | Black or African American | 176.4 | 0.84 | 0.01 | 5 | GPH |
| OXPC011 | Blood | White | 53.4 | 0.89 | 0.00 | - | GPH |
| OXPC011 | Tissue | White | 20.4 | 0.52 | 0.65 | 5 | GPH |
| OXPC012 | Blood | White | 66.0 | 0.71 | 0.00 | - | H |
| OXPC014 | Blood | White | 12.1 | 0.68 | 0.00 | - | GPH |
| OXPC014 | Tissue | White | 15.0 | 0.68 | 0.11 | 3 | GPH |
| OXPC017 | Blood | White | 24.2 | 0.71 | 0.00 | - | GPH |
| OXPC017 | Tissue | White | 30.6 | 0.79 | 0.01 | 3 | GPH |
| OXPC018 | Blood | Asian | 14.8 | 1.01 | 0.00 | - | GPH |
| OXPC018 | Tissue | Asian | 25.3 | 0.74 | 0.00 | 8 | GPH |

1. Age of block inferred as the difference in years from DNA extraction (2021) to the year of the diagnosis.
2. Analysis type sample was used in, which was one of:

GPH: Genome wide, PRS and HLA

H: HLA only (for samples without both tissue and blood)

### Table S2. PRS description.

| **Phenotype** | **# Variants** | **# SNPs** | **# SNPs imputed** | **# SNPs high-quality** | **PGSID** | **Publication source** |
| --- | --- | --- | --- | --- | --- | --- |
| Bladder cancer | 15 | 15 | 14 | 13 | PGS000071 | https://doi.org/10.1038/s41467-021-21288-z |
| Breast cancer  *(Graff et al., 2021)* | 187 | 172 | 167 | 153 | PGS000072 | https://doi.org/10.1038/s41467-021-21288-z |
| Cervical cancer | 10 | 9 | 9 | 9 | PGS000073 | https://doi.org/10.1038/s41467-021-21288-z |
| Colorectal cancer | 103 | 103 | 101 | 90 | PGS000074 | https://doi.org/10.1038/s41467-021-21288-z |
| Endometrial cancer | 9 | 9 | 9 | 9 | PGS000075 | https://doi.org/10.1038/s41467-021-21288-z |
| Kidney cancer | 19 | 19 | 16 | 16 | PGS000076 | https://doi.org/10.1038/s41467-021-21288-z |
| Lymphocytic leukemia | 75 | 75 | 69 | 65 | PGS000077 | https://doi.org/10.1038/s41467-021-21288-z |
| Lung cancer | 109 | 109 | 100 | 85 | PGS000078 | https://doi.org/10.1038/s41467-021-21288-z |
| Melanoma | 24 | 24 | 22 | 21 | PGS000079 | https://doi.org/10.1038/s41467-021-21288-z |
| Non-Hodgkin's lymphoma | 19 | 18 | 17 | 16 | PGS000080 | https://doi.org/10.1038/s41467-021-21288-z |
| Oral cavity and pharyngeal cancers | 14 | 13 | 8 | 8 | PGS000081 | https://doi.org/10.1038/s41467-021-21288-z |
| Ovarian cancer | 36 | 32 | 30 | 27 | PGS000082 | https://doi.org/10.1038/s41467-021-21288-z |
| Pancreatic cancer | 22 | 22 | 22 | 18 | PGS000083 | https://doi.org/10.1038/s41467-021-21288-z |
| Prostate cancer | 161 | 152 | 146 | 136 | PGS000084 | https://doi.org/10.1038/s41467-021-21288-z |
| Testicular cancer | 52 | 52 | 49 | 45 | PGS000086 | https://doi.org/10.1038/s41467-021-21288-z |
| Thyroid cancer | 12 | 11 | 11 | 10 | PGS000087 | https://doi.org/10.1038/s41467-021-21288-z |
| Type 1 Diabetes (T1D) | 825 | 825 | 818 | 747 | PGS001817 | https://doi.org/10.1016/j.ajhg.2021.11.008 |
| Type 2 Diabetes (T2D) | 384 | 384 | 378 | 321 | PGS000832 | https://doi.org/10.1038/s41588-021-00948-2 |
| High lipoprotein density (HLD) | 303 | 302 | 265 | 241 | PGS000845 | https://doi.org/10.1038/s41588-021-00948-2 |
| Body mass index (BMI) | 122 | 122 | 122 | 117 | PGS000841 | https://doi.org/10.1038/s41588-021-00948-2 |
| Cardiovascular disease (CVD) | 330 | 329 | 224 | 212 | PGS000863 | https://doi.org/10.1038/s41588-021-00948-2 |
| Ulcerative colitis (UC) | 179 | 165 | 147 | 132 | PGS001306 | https://doi.org/10.1101/2021.09.02.21262942 |
| Breast cancer  (Zhang et al., 2015) | 330 | 270 | 270 | 241 | PGS000212 | https://doi.org/10.1038/s41588-020-0609-2 |
| Breast cancer  (Mavaddat et al., 2019) | 313 | 265 | 256 | 230 | [PGS00000](https://www.pgscatalog.org/score/PGS000001)4 | https://doi.org/10.1016/j.ajhg.2018.11.002 |

###

### Table S3. DCIS cohort technical characteristic description.

| **Patient** | **Input DNA (ng)** | **Mean coverage** | **CNA burden** | **Age of block (yr)^1^** | **Passed QC?** |
| --- | --- | --- | --- | --- | --- |
| OXPA002 | 68.4 | 0.93 | 0.05 | 18 | Yes |
| OXPA003 | 19.6 | 1.07 | 0.04 | 21 | Yes |
| OXPA006 | 106.8 | 0.96 | 0.06 | 14 | Yes |
| OXPA020 | 69.6 | 1.07 | 0.02 | 22 | Yes |
| OXPA021 | 99 | 1.11 | 0.03 | 21 | Yes |
| OXPA028 | 29.64 | 0.84 | 0.05 | 16 | Yes |
| OXPA032 | 38.4 | 1.09 | 0.04 | 15 | Yes |
| OXPA033 | 300 | 0.96 | 0.08 | 15 | Yes |
| OXPA036 | 12.7 | 0.88 | 0.07 | 14 | Yes |
| OXPA044 | 65.4 | 1.10 | 0.03 | 11 | Yes |
| OXPA064 | 15 | 1.12 | 0.04 | 7 | Yes |
| OXPA092 | 10.3 | 0.49 | 0.02 | 14 | Yes |
| OXPA146 | 24.06 | 0.81 | 0.08 | 25 | Yes |
| OXPA147 | 300 | 0.74 | 0.04 | 25 | Yes |
| OXPA150 | 30.9 | 0.92 | 0.04 | 24 | Yes |
| OXPA151 | 64.8 | 0.79 | 0.37 | 24 | Yes |
| OXPA153 | 201 | 0.96 | 0.05 | 24 | Yes |
| OXPA156 | 19.9 | 0.83 | 0.03 | 24 | Yes |
| OXPA161 | 15.4 | 0.95 | 0.04 | 24 | Yes |
| OXPA166 | 300 | 0.91 | 0.04 | 24 | Yes |
| OXPA172 | 28.02 | 1.16 | 0.03 | 23 | Yes |
| OXPA179 | 102 | 0.79 | 0.04 | 23 | Yes |
| OXPA182 | 31.2 | 0.83 | 0.28 | 23 | Yes |
| OXPA185 | 52.2 | 0.89 | 0.04 | 23 | Yes |
| OXPA246 | 109.2 | 1.16 | 0.04 | 20 | Yes |
| OXPA267 | 204 | 1.01 | 0.07 | 19 | Yes |
| OXPA295 | 25 | 1.65 | 0.02 | 18 | Yes |
| OXPA347 | 40.2 | 1.79 | 0.05 | 16 | Yes |
| OXPA392 | 6.6 | 1.36 | 0.04 | 15 | Yes |
| OXPA445 | 7.5 | 1.12 | 0.02 | 14 | Yes |
| OXPA501 | 119.4 | 0.93 | 0.04 | 13 | Yes |
| OXPA508 | 25.5 | 0.67 | 0.05 | 13 | Yes |
| OXPA527 | 48.9 | 0.68 | 0.10 | 13 | Yes |
| OXPA530 | 14.4 | 0.74 | 0.30 | 13 | Yes |
| OXPA540 | 72.6 | 0.96 | 0.05 | 12 | Yes |
| OXPA644 | 30.9 | 0.59 | 0.04 | 10 | Yes |
| OXPA007 | 82 | 0.15 | N/A | 11 | No (Low cov.) |
| OXPA035 | 17.5 | 0.31 | N/A | 14 | No (Low cov.) |
| OXPA619 | 35.2 | 0.31 | N/A | 11 | No (Low cov.) |
| OXPA066 | 300 | 0.34 | N/A | 7 | No (Low cov.) |
| OXPB024 | 53.7 | 0.47 | N/A | 6 | No (Low cov.) |
| OXPA025 | 300 | 0.49 | N/A | 17 | No (Contam.) |
| OXPA574 | 50.1 | 0.80 | N/A | 12 | No (Contam.) |
| OXPA005 | 18.6 | 0.81 | N/A | 15 | No (Contam.) |
| OXPA269 | 11.9 | 0.91 | N/A | 19 | No (Contam.) |
| OXPA165 | 81.6 | 0.92 | N/A | 24 | No (Contam.) |
| OXPA040 | 300 | 0.93 | N/A | 13 | No (Contam.) |
| OXPA169 | 300 | 0.97 | N/A | 24 | No (Contam.) |
| OXPB009 | 224.4 | 0.99 | N/A | 7 | No (Contam.) |
| OXPA029 | 170.4 | 1.04 | N/A | 16 | No (Contam.) |

1. Age of block inferred as the difference in years from DNA extraction (2021) to the year of the diagnosis.

###

### Table S4. DCIS cohort covariate association with patient outcome.

| **Clinical feature** |  | **No BCSE^2^**  **(N=14)** | **BCSE**  **(N=22)** | **Significance^3^** |
| --- | --- | --- | --- | --- |
| **Grade^1^** | Low | 36% (5) | 41% (9) | *p=0.59* |
|  | Intermediate | 50% (7) | 27% (6) |  |
|  | High | 7% (1) | 9% (2) |  |
| **ER status^1^** | **+** | 71% (10) | 77% (17) | *p=0.39* |
|  | **-** | 7% (1) | 0% (0) |  |
| **Ethnicity^1^** | Hispanic | 14% (2) | 9% (2) | *p=0.63* |
|  | Non-Hispanic | 86% (12) | 91% (20) |  |
| **Race** | Asian | 14% (2) | 9% (2) | *p=0.63* |
|  | White | 86% (12) | 91% (20) |  |
| **Pathologic size (cm)**^1^ |  | 1.35 | 0.85 | *p=0.05* |
| **Age at diagnosis (yrs)** |  | 56.3 | 60.1 | *p=0.27* |

1. Missing values not represented here, but can be found in Table S2.
2. BCSE: Breast cancer subsequent event.
3. P-values were computed using Fisher Exact test for ER status, Race, Ethnicity and Chi-square test for Grade. For continuous features, size and age, Mann-Whitney U test was used to compare groups.

### 
